# Panobinostat (LBH589) combined with AM1241 induces cervical cancer cell apoptosis through autophagy pathway

**DOI:** 10.1186/s40360-023-00686-7

**Published:** 2023-09-22

**Authors:** Bo Sheng, Wenwen Wang, Dongyue Xia, Xiangdong Qu

**Affiliations:** 1 Department of Obstetrics and Gynecology, Taizhou Central Hospital, Taizhou University Hospital, 999 Donghai Avenue, High-Tech Zone, Taizhou, 318000 China; 2 Department of Hematology and Oncology, Taizhou Central Hospital, Taizhou University Hospital, Taizhou, 318000 China

**Keywords:** Panobinostat (LBH589), Autophagy, Cervical cancer, Cell apoptosis, CB2 receptor agonist

## Abstract

**Purpose:**

The study aims to investigate the apoptotic effects of combining LBH589 and AM1241 (a selective CB2 receptor agonist) on cervical cancer cells and elucidating the mechanism of this combined therapy, which may provide innovative strategies for treating this disease.

**Methods:**

The viability of the cervical cancer cells was measured by cell counting kit-8 (CCK-8) assay, and the synergistic effect was analyzed using SynergyFinder. Cell proliferation was tested by cell cloning. The apoptosis and reactive oxygen species (ROS) production in cervical cancer cells were analyzed by flow cytometry. Western blot and quantitative real-time PCR (qRT-PCR) were employed to determine changes in protein and gene levels of pathway-related factors.

**Results:**

By the results of cytotoxicity assay, SiHa cells were selected and treated with 0.1 μM LBH589 and 4 μM AM1241 for 24 h for subsequent experiments. The combination of both was synergistic as determined by bliss, ZIP, HSA and LOEWE synergy score. Plate cloning results showed that LBH589 combined with AM1241 inhibited the proliferation of cervical cancer cells compared to individual drug. Additionally, compared with LBH589 alone, the combination of LBH589 and AM1241 induced autophagy by increasing LC3II/LC3I and decreasing P62/GAPDH, leading to a significantly higher rate of apoptosis. Pharmacological inhibition of also inhibited apoptosis. Consistently, we found that the endoplasmic reticulum, DNA damage repair pathway were induced after co-administration. Furthermore, cellular ROS increased after co-administration, and apoptosis was inhibited by the addition of ROS scavenger.

**Conclusion:**

LBH589 combined with AM1241 activated the endoplasmic reticulum emergency pathway, DNA damage repair signaling pathway, oxidative stress and autophagy pathway, ultimately promoting the apoptosis of cervical cancer cells. These findings suggest that the co-administration of LBH589 and AM1241 may be a new treatment plan for the treatment of cervical cancer.

**Supplementary Information:**

The online version contains supplementary material available at 10.1186/s40360-023-00686-7.

## Introduction

Cervical cancer is the fourth most common malignancy among women, triggered by a high-risk human papillomavirus (hrHPV) infection. With a tendency for decreasing average age of onset among female patients, cervical cancer is a growing concern [1]. Recent advancements in cervical cancer biology have revealed the prevalence of epigenetic alterations, particularly histone modifications, in cervical carcinogenesis and metastasis [2]. Among them, histone modifications are essential for regulating gene expression through chromatin modifications, [3] such as histone acetylation which is a process regulated by histone deacetylase (HDAC) and histone acetyltransferase (HAT). Studies have found that HDAC is overexpressed in a range of malignancies, including cervical cancer, making HDAC a potential target for cancer therapy [4].

Antineoplastic LBH589 is an oral histone deacetylase inhibitor that can change the expression of genes by causing acetylation of histone lysine tails in chromatin. It has been found to have good potential in anti-cancer in recent years, such as the ability to inhibit the growth and metastasis of hepatocellular carcinoma, [5] anti-ovarian cancer activity in vivo and in vitro*, * [6] and the ability to inhibit breast tumor growth and metastasis [7]. Besides, Liu et al. identified LBH589 as a potential drug for use in patients with high-risk cervical cancer through interaction analysis between risk profile and drug response [8]. Despite its potential, phase II trials using HDACi against solid tumors have shown limited success, with only a small number of patients achieve complete remission [4]. However, the combination of LBH589 and topoisomerase inhibitors, such as topotecan or etoposide, has been shown to induce strong cell death in cervical cancer-derived cells by activating the intrinsic apoptotic pathway [9]. This suggests that the combination of HDACi and another apoptosis-inducing tumor suppressor may be an efficient cervical cancer treatment plan.

AM1241 is a potent, classical, selective cannabinoid receptor 2 (CB2) receptor agonist. The over-expression of human CB2 promoted apoptosis of cervical cancer cells via up-regulating the Bax, Bad expressions and down-regulating the Bcl-2 expression [10]. Recent studies have indicated that AM1241 significantly suppresses p53-dependent apoptosis by stimulating production of IL-10 [11]. Combining the properties of LBH589 and AM1241, LBH589 may hold promise in producing unexpected anti-cervical cancer activity in combination with AM1241. However, the function of LBH589 when used in conjunction with AM1241 for cervical cancer remains uncertain.

Therefore, we analyzed the role of LBH589 and AM1241 in inducing apoptosis in cervical cancer cells in this study, respectively, and examined the role of LBH589 combined with AM1241 in cervical cancer cell apoptosis and related mechanisms.

## Materials and methods

### Cell culture

Hela and SiHa cell lines were obtained from Shanghai Qi Da Biological Technology Co., Ltd. The cells were maintained in Dulbecco's modified Eagle medium (DMEM, Gibco Invitrogen Corporation, Carlsbad, CA), supplemented with 10% fetal bovine serum (FBS) (Bayotime, Shanghai, China) and 1% antibiotics (100 units/mL penicillin and 100 μg/mL streptomycin (Invitrogen-Life Technologies, Carlsbad, CA)), in a humidified atmosphere of 5% CO_2_ at 37°C.

### Cell viability

The effect of LBH589 (MedChemExpress, USA) and AM1241 (Sigma-Aldrich, USA) on the cellular activity of HeLa and SiHa cells was determined using the cell counting kit-8 (CCK-8) assay. Various concentrations of LBH589 (0, 0.1, 0.25, 0.5, 1, and 2 μM) or AM1241 (0, 1, 4, 10, 40, and 100 μM) were administered to the cells for different time periods (24 and 48 h) (*n* = 6). Cell suspensions (100 μl/well) were inoculated in 96-well plates and pre-cultured for 4 h at 37°C with 5% CO_2_. Additionally, co-treat cells with LBH589 and AM1241 to evaluate their synergistic effects using the SynergyFinder website ( http://synergyfinder.org/#!/) (*n* = 5). After treatment, 10 μl of CCK-8 solution (Sigma-Aldrich, USA) was added to each well and incubated for 1 h protected from light. Then the absorbance was measured at 450 nm using a precise microplate reader (MolecularDevices, Sunnyvale, CA).

### Colony formation assay

The Hela and SiHa cells were distributed into control group, 0.1μM LBH589 group, 4 μM AM1241 group and 0.1μM LBH589 + 4 μM AM1241 group. The subsequent experiments were conducted in accordance with the aforementioned grouping. Cells were seeded in the culture plates(1.5 × 10^3^/well) and incubated for a period of 1 week to form colonies before the cells were stained with crystal violet solution (*n* = 5).

### Western blot analysis

The extracted proteins from treated cells were analyzed using SDS-PAGE (Beyotime Biotechnology, Shanghai, China) and transferred to a PVDF membrane (Beyotime Biotechnology). The membrane was blocked and then incubated overnight at 4°C with primary antibodies against activator of transcription factor 4 (ATF4), ATF6, eukaryotic initiation factor 2α (EIF2α), phosphorylated EIF2α (p-EIF2α), X-box binding protein 1 (XBP-1), C/EBP homologous protein (CHOP), γ-H2AX, light chain 3 (LC3), P62, cleaved-caspase3, cleaved-PARP, and glyceraldehyde-3-phosphate dehydrogenase (GAPDH), followed by incubation with secondary antibodies. The antibodies utilized in the analysis were procured from Proteintech, USA. Analysis of protein bands was conducted using Image J (Bio-Rad, USA).

### Quantitative Real-time Polymerase Chain Reaction (qRT-PCR)

Treated cells were lysed using Trizol and then total RNA was obtained. RNA concentrations were determined spectrophotometrically. The total RNA was then added to nuclease-free PCR tubes on ice along with nuclease-free water, TransScript All-in-One SuperMix for qPCR, and gDNA Remover. The samples were incubated at 42°C for 15 min, and then heated at 85°C for 5 s to inactivate TransScript RT/RI and gDNA Remover. The resulting cDNA was diluted tenfold and used as a template for qPCR. For each reaction, 2 µl of the cDNA solution was added to 10 µl reaction of SybrGreen qPCR Master Mix (TAKARA), 0.4 µl (1 µM) of both forward and reverse primers, and 7.2 µl of nuclease-free water. qRT-PCR primers for LC3: Forward 5’-GAGTTACCTCCCGCAGCC-3’, Reverse 5’-TTACAGCGGTCGGCGAAG-3’. qRT-PCR primers for P62: Forward 5’-GATTCGCCGCTTCAGCTTCT-3’, Reverse 5’-CCCGTCCTCATCGCGGTA-3’. Reactions were performed in an ABI Stepone plus real-time PCR instrument. All PCR cycles began with denaturation for 3 min at 95°C, followed by 40 cycles of 7 s for 95°C, 10 s for 57 °C and 15 s for 72°C. Relative mRNA expression was calculated using the 2^−ΔΔCt^ method.

### Cell apoptosis assay

The rate of apoptosis was quantified using the Annexin V FITC Apoptosis Kit (BD Biosciences, USA). Briefly, upon digestion, resuspended samples were labeled with Annexin V fluorescein isothiocyanate and propidium iodide and incubated for 15 min prior to being subjected to flow cytometry (BD Biosciences, USA). The resulting data were analyzed using FlowJo (BD Pharmingen, USA). The autophagy inhibitor, 3-methyladenine (3-MA), and the ROS scavenger, N-acetyl-L-cysteine (NAC), were obtained from Sigma-Aldrich (USA).

### Measurement of ROS generation

The cells were pre-incubated at 37°C for 2 h, and subsequently treated with the drug for the specified period of time. Subsequently, the cells were resuspended in medium and incubated with DCFH-DA at 37°C for 20 min. The resulting fluorescence intensity was assessed using a flow cytometer (BD Biosciences, USA).

### Statistical analysis

Statistical analysis of result was performed using one-way ANOVA by GraphPad Prism v8.0.2. All data are given as mean ± standard deviation. A *p* < 0.05 value was considered significant. Each experiment was repeated at least thrice with three replicates.

## Results

### LBH589 combination with AM1241 inhibited the proliferation of cervical cancer cells

**Fig. 1:**
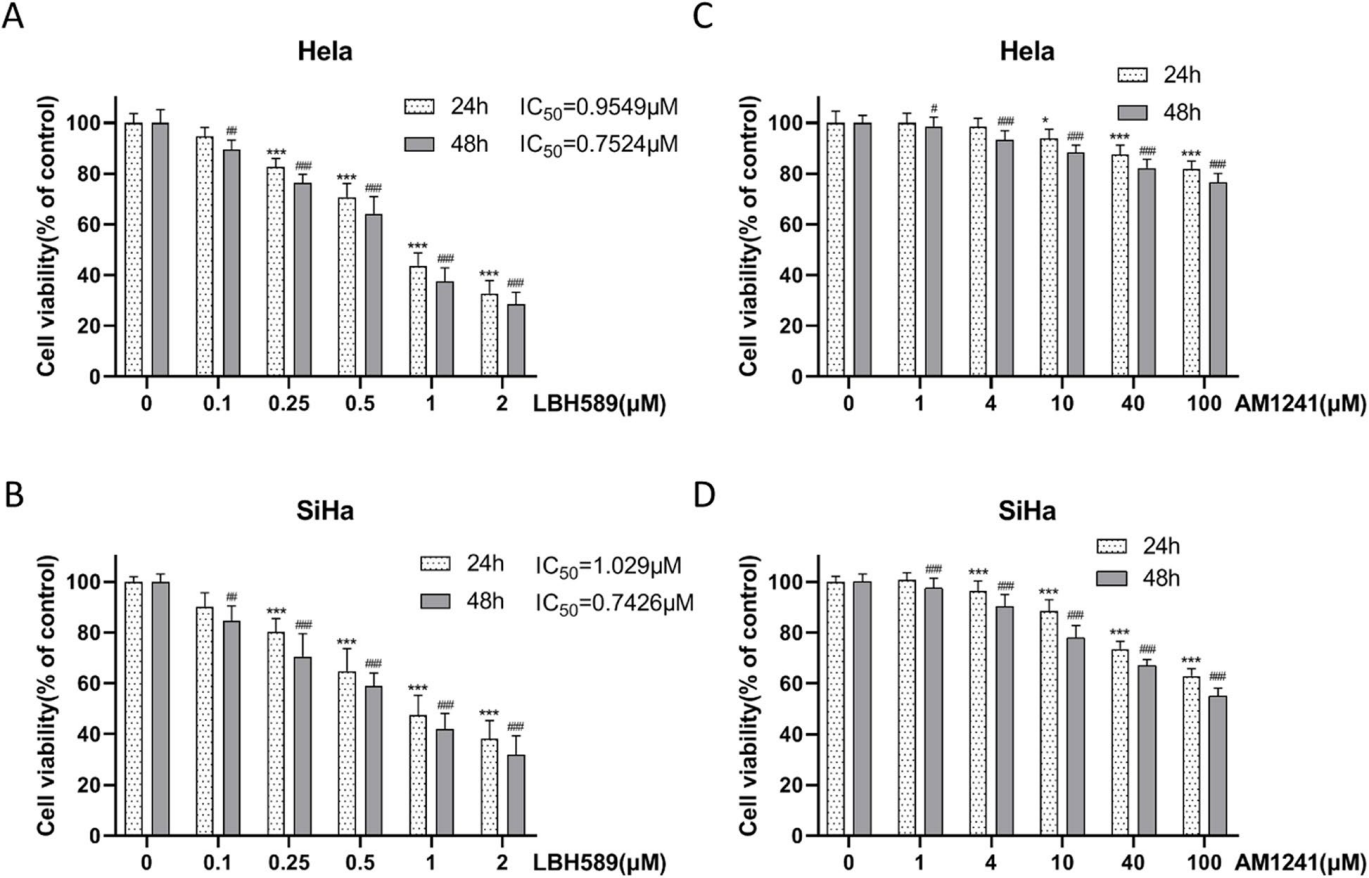
The appropriate treating concentration of LBH589 and AM1241 on cervical cancer cells. **A** LBH589 was applied to HeLa cells at various concentrations in an effort to find the appropriate amount (*n* = 6). **B** LBH589 was applied to SiHa cells at various concentrations in an effort to find the appropriate amount. Compared with 0 μM LBH589 in 24h, ^***^P < 0.001; compared with 0 μM LBH589 in 48h, ^##^*P* < 0.01, ^###^*P* < 0.001 (*n* = 6). **C** AM1241 was applied to HeLa cells at various concentrations in an effort to find the appropriate amount (*n* = 6). **D** AM1241 was applied to SiHa cells at various concentrations in an effort to find the appropriate amount (*n* = 6). Compared with 0μM AM1241 in 24h, ^*^P < 0.05, ^***^P < 0.001; compared with 0 μM AM1241 in 48h, ^#^*P* < 0.05, ^###^*P* < 0.001

Prior to the experiment, both Hela and SiHa cervical cancer cell lines were subjected to various concentrations of LBH589 (0, 0.1, 0.25, 0.5, 1 and 2 μM) and AM1241 (0, 1, 4, 10, 40, 100 μM) for 24 h and 48 h to evaluate the cell viability. The results revealed that when treated with 0.1 μM LBH589 for 24 h, the viability of Hela cells remained relatively stable (Fig. 1A and B). Similarly, the viability of Hela and SiHa cells remained stable after being treated with 4 μM AM1241 for 24 h (Fig. 1C and D). Therefore, 0.1 μM LBH589 and 4 μM AM1241 and 24 h treatment time were selected as the conditions for the follow-up experiments.

**Fig. 2:**
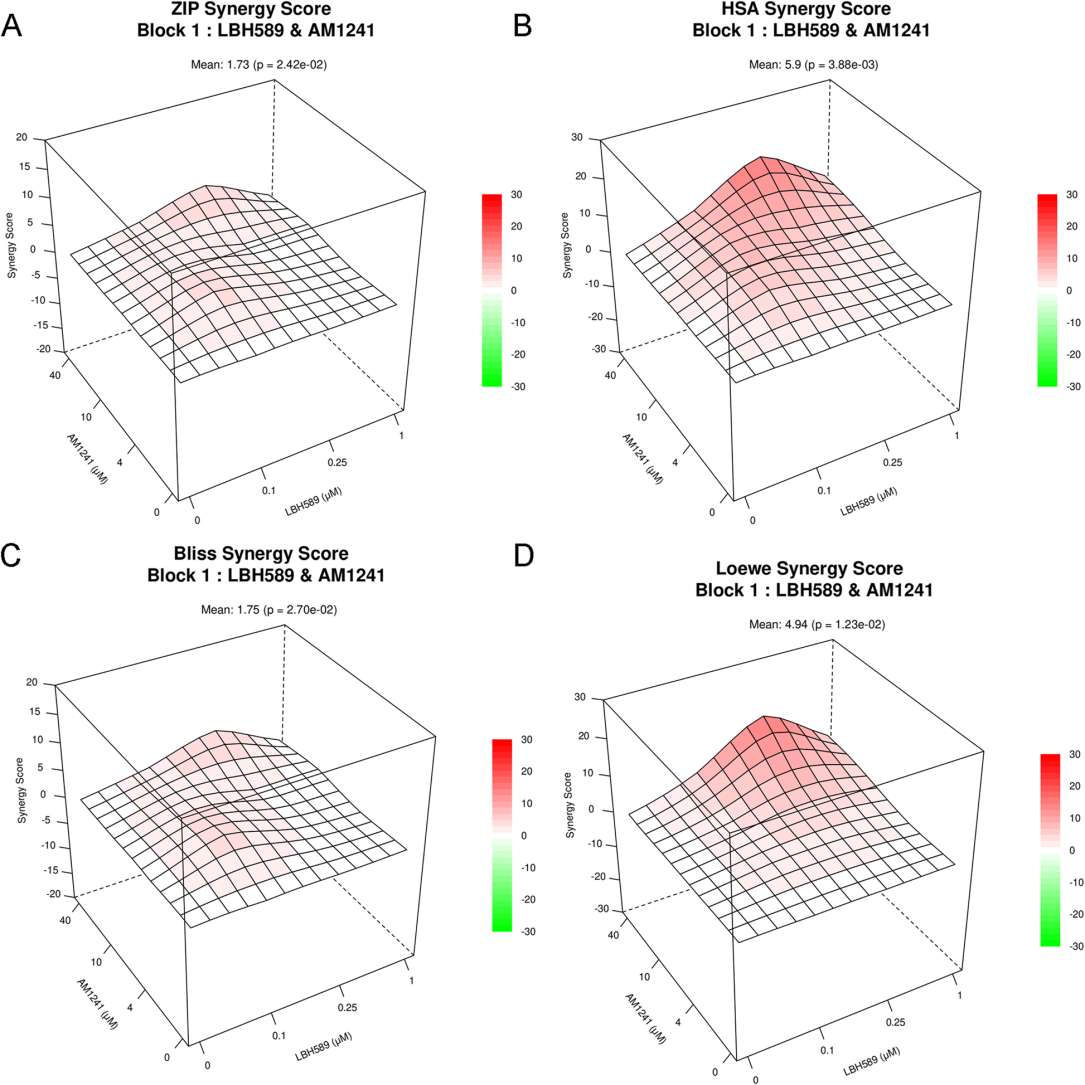
Synergistic effect chart of AM1241 and LBH 589 combined treatment on Hela cervical cancer cells. Synergistic effect charts of Hela cervical cancer cells were calculated using **A** ZIP, **B** HSA, **C** Bliss, and **D** Loewe reference models. These data were obtained using the SynergyFinder software. The synergistic effect is manifested as a ZIP synergy score of more than 1, and Loewe, Bliss, and HSA synergistic scores of more than 0 (*n* = 5)

The potential for synergistic cytotoxic activity between LBH589 and AM1241 was assessed using SynergyFinder software based on four reference models: ZIP, HSA, Bliss, and Loewe [12]. Upon analyzing the synergistic graph of LBH589 and AM1241, it was observed that the drugs in this combination exhibit a synergistic effect across the four models. The corresponding synergy scores for these models were 1.73, 5.9, 1.75, and 4.94 (Fig. 2 and S1).

**Fig. 3:**
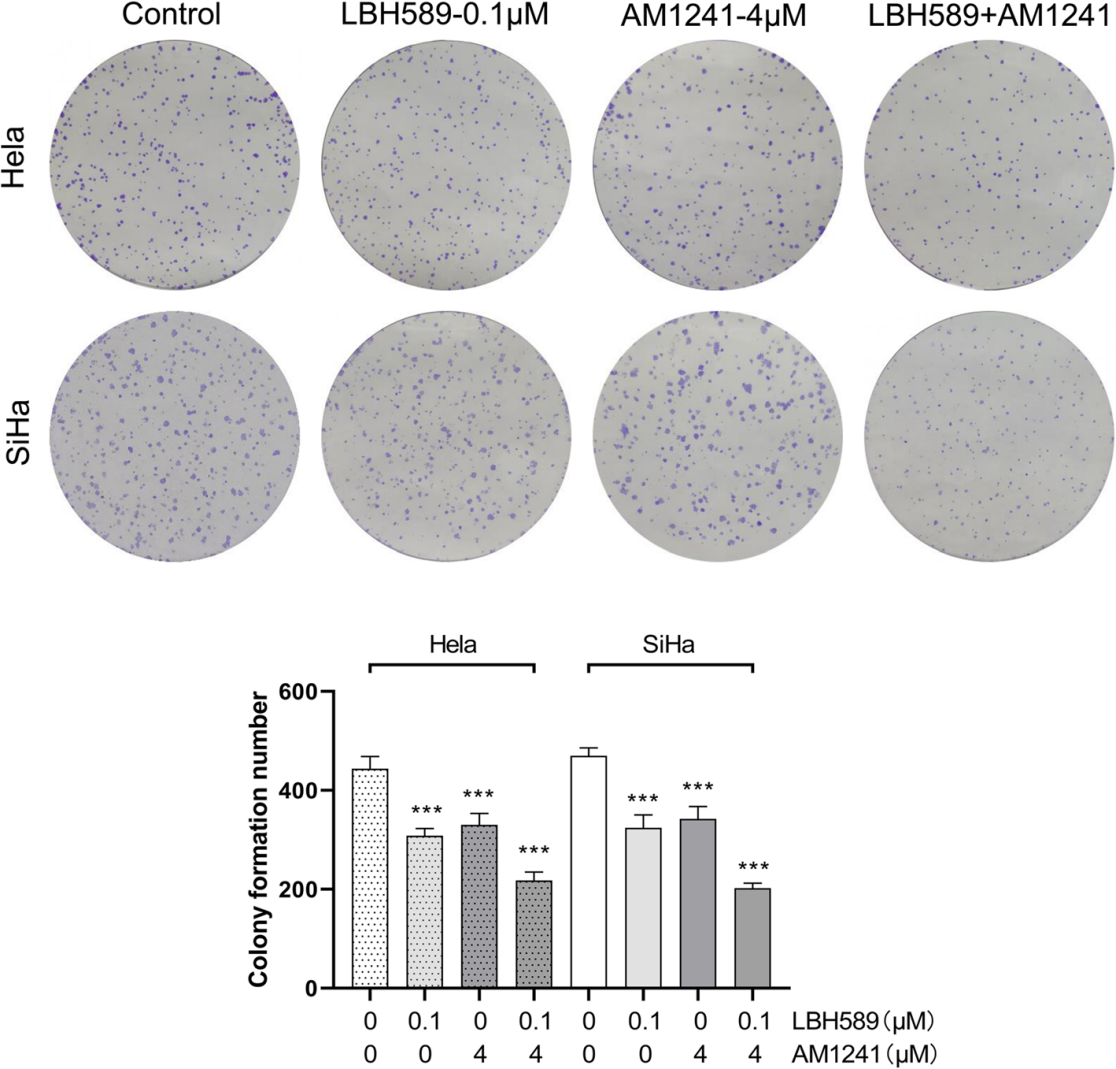
The effect of different concentrations of LBH589 or AM1241 on the proliferation of cervical cancer cells. The impact of LBH589 and AM1241, either alone or in conjunction, on the proliferation of HeLa and SiHa cells was assessed via colony formation assay (*n* = 3). Compared with 0 μM AM1241 + 0 μM LBH589, ^***^P < 0.001

The results of cell cloning experiments showed that both of LBH589 and AM1241 inhibited the proliferation of Hela cells and SiHa cells, while LBH589 combined with AM1241 produced a stronger inhibition of proliferation (Fig. 3). Given that the IC_50_ value for LBH589 on SiHa and Hela cells was similar, and the IC_50_ value for AM1241 was significantly lower on SiHa cells, SiHa was chosen for further experimentation.

### LBH589 combination with AM1241 activates endoplasmic reticulum (ER) stress pathway and DNA damage repair (DDR) signaling pathway to promote apoptosis in cervical cancer cells

**Fig. 4:**
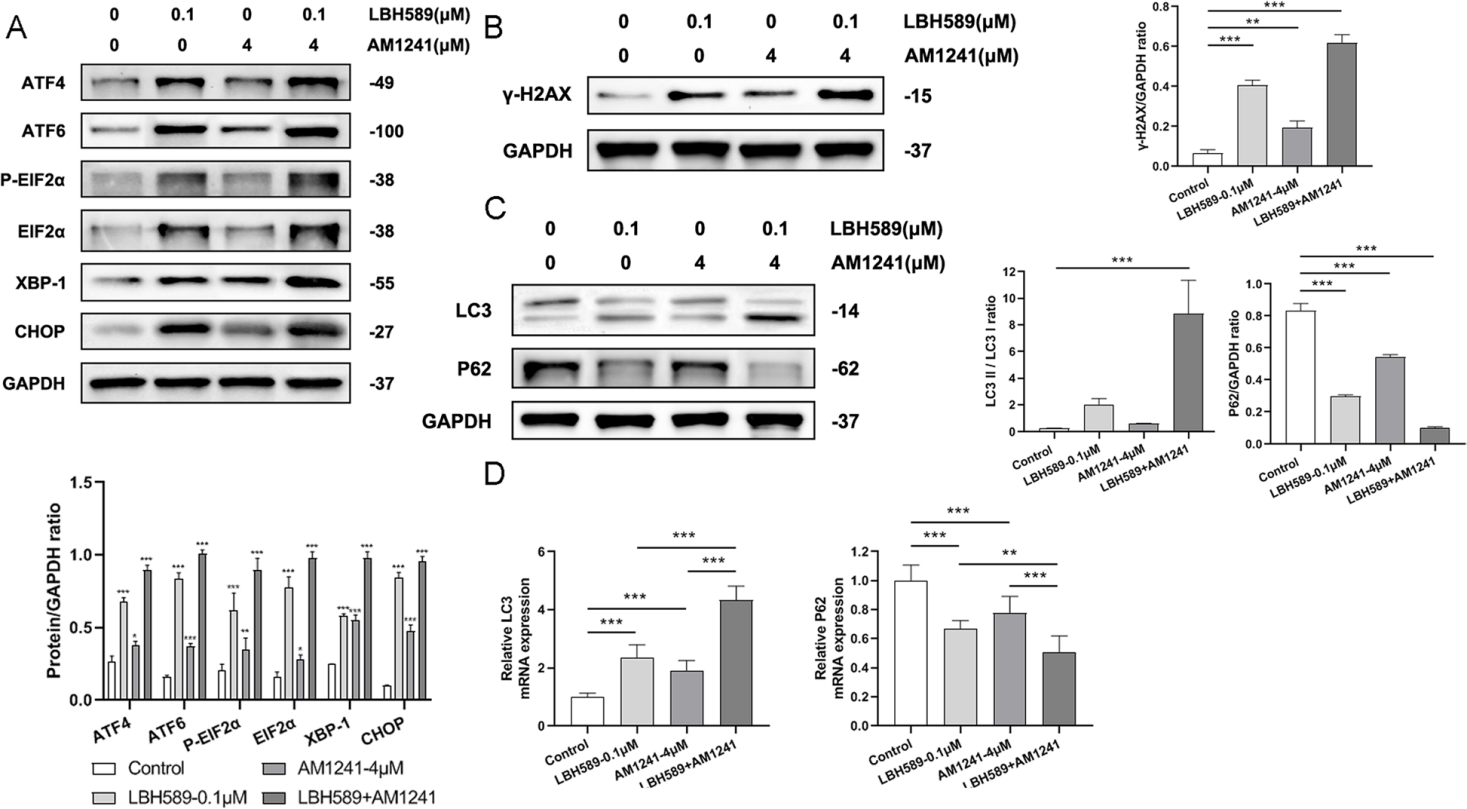
The influence of LBH589 combined with AM1241 on the channels. LBH589 combined with AM1241 activated **A** endoplasmic reticulum stress pathway (*n* = 3), **B** DDR signaling pathway (*n* = 3) and **C** and **D** autophagy (*n* = 3). Compared with the control group, ^*^P < 0.05, ^**^P < 0.01, ^***^P < 0.001

To probe the means by which the combination of LBH589 and AM1241 inhibited the proliferation of cervical cancer cells, an investigation was carried out on the activation of the ER stress, DDR, and autophagy signaling pathways. The results indicated that the addition of LBH589 or AM1241 increased the expression of ATF4, ATF6, EIF2α, XBP-1, CHOP, besides, the expression of the above ER stress pathway-related factors was significantly increased when the cells were treated with the combination of LBH589 and AM1241, indicating that the ER stress pathway was activated by the combination of LBH589 and AM1241 (Fig. 4A). Additionally, both LBH589 and AM1241 elicited an upregulation of γ-H2AX expression, and when used in conjunction, γ-H2AX expression was significantly augmented, thus activating the DDR signaling pathway (Fig. 4B). In agreement, we observed an increase in LC3 expression and a decrease in P62 expression at both protein and mRNA levels upon the administration of either drug, and when both drugs were combined, the enhancement of LC3 expression and reduction of P62 expression were notably amplified, attesting to the activation of the autophagy pathway (Fig. 4C and D).

### LBH589 combined with AM1241 promoted cervical cancer cell apoptosis and oxidative stress

**Fig. 5:**
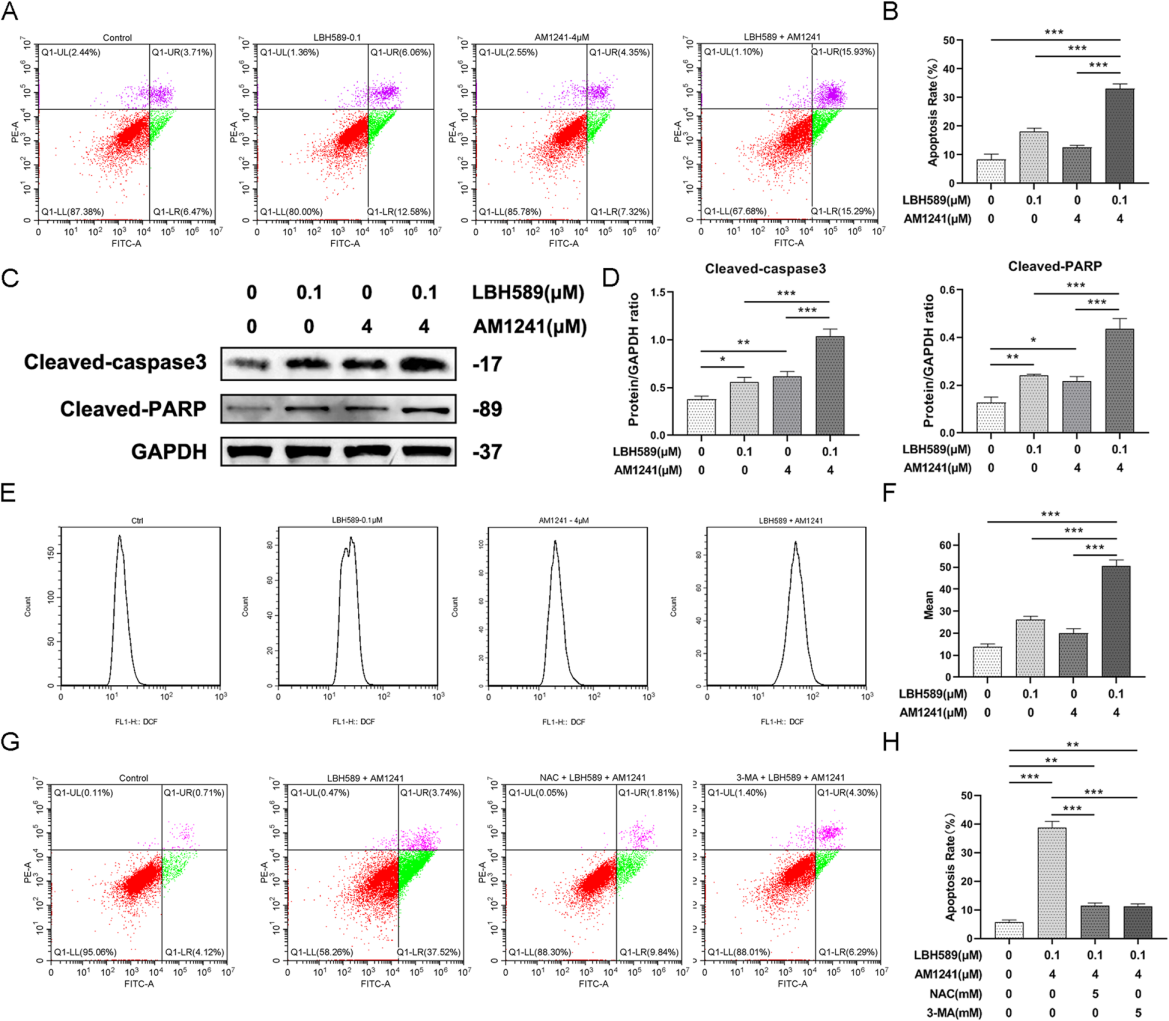
The effect of LBH589 combined with AM1241 on apoptosis and oxidative stress. **A**-**D** Apoptosis detection (*n* = 3). ^*^P < 0.05, ^**^P < 0.005, ^***^P < 0.001. **E** and **F** The effect of LBH589 combined with AM1241 monotherapy on ROS (*n* = 3), ^***^P < 0.001. **G** and **H** To detect the effect on apoptosis after adding ROS scavenger NAC or autophagy inhibitor 3-MA (*n* = 3), ^**^P < 0.01, ^***^P < 0.001

The addition of LBH589 or AM1241 resulted in a significant increase in apoptosis, as evidenced by the flow cytometric assay (Fig. 5A and B). Besides, the apoptosis-related protein cleaved-caspase3 and cleaved-PARP upregulated upon treatment with LBH589 or AM1241(Fig. 5C and D). Notably, the combined use of LBH589 and AM1241 resulted in a higher apoptosis rate compared to the use of either agent alone. Upon further investigation into the effect of combined dosing of LBH589 and AM1241 on cervical cancer cells, we observed a significant increase in ROS in SiHa cells (Fig. 5E and F). Apoptosis of SiHa cells was suppressed by the addition of the ROS scavenger NAC, with the effect of NAC being comparable to that of adding the autophagy inhibitor 3-MA. This indicates that LBH589 combined with AM1241 promotes apoptosis of cervical cancer cells by activating autophagy and increasing ROS accumulation (Fig. 5G and H).

## Discussion

The incidence of cervical cancer ranks fourth globally, claiming the lives of nearly 300,000 individuals annually, with approximately 600,000 new cases diagnosed each year [1]. In our study, we demonstrated a synergistic anti-cancer effect in the combination of LBH589 and AM 1241. Apoptosis of cervical cancer cells could be induced and enhanced by autophagy activation, DDR signaling pathway activation and endoplasmic reticulum stress, all of which could be induced by LBH589 and AM1241. The use of LBH589 and AM1241 to induce these pathways could potentially be a valuable addition to the current cervical cancer treatment regimen. Further research in this area could provide more insights into the underlying mechanisms of cervical cancer and aid in the development of more effective treatment options.

Activation of autophagy has emerged as a promising treatment strategy in combination with existing cancer therapies [13]. Studies have shown that autophagy can be induced by regulating targeted genes (such as Bcl-2, THBS2) and pathways (such as Wnt signaling pathway) to promote apoptosis in cervical cancer cell [14–16]. LBH589, as the most effective HDACi in clinical development, can promote tumor cell apoptosis by inducing autophagy. Fazio et al. stated that the combined use of LBH589 and tamoxifen induces autophagy and leads to the death of liver cancer cells [17]. In recent years, studies have found that CB2 plays a protective role in various pathological conditions by activating autophagy, [18]. However, no studies have examined the effect of CB2 receptor agonists such as AM1241 on cervical cancer cells. Liu et al. demonstrated that AM1241 induced autophagy through activation of the Pink1/Parkin pathway, attenuating myocardial ischemia–reperfusion injury in rats [19]. In our study, both of LBH589 and AM1241 induced autophagy in SiHa cells. In addition, AM1241 and LBH589 work better together than either drug alone in inducing autophagy in SiHa cells. After blocking autophagy, cells apoptosis was inhibited, instructing us that LBH589 and AM1241 are able to promote apoptosis by inducing autophagy.

In addition to autophagy activation, apoptotic signaling can be generated from cell membrane, mitochondria and ER. Among them, ER stress is considered to be the key mechanism of many pathological processes and diseases [20]. It has been reported that inhibiting the activation of ER stress pathway and autophagy through drugs may enhance cytotoxicity [21]. Our research found that the combined use of LBH589 and AM1241 induced the activation of the ER stress pathway, thereby promoting apoptosis in cervical cancer cells.

Studies have shown that radiation-induced autophagy can directly or indirectly damage DNA, thereby activating the DDR signaling pathway and causing damage to the extranuclear target ER [22]. DDR, a network of cellular pathways, prevents the reproduction of damaged DNA to maintain genome integrity. Mutations or loss of function of the components of these pathways is a carcinogenic step [23]. Studies have shown that the activation of DDR protein is necessary to complete the virus life cycle and is closely related to the replication and transformation of HPV [24]. In our investigation, it was observed that the combination of LBH589 and AM1241 not only activates the ER stress pathway, but also triggers the DDR signaling pathway. The interplay between DDR and autophagy holds great promise in developing novel and efficacious strategies for treating cancer.

Indeed, as has been well established, the formation of autophagy is intricately linked to the impact of oxidative stress [25]. In our study, we observed that compared to the use of either agent alone, the combination of LBH589 and AM1241 led to a substantial increase in cellular ROS levels. Further, we observed that the addition of a ROS scavenger resulted in a significant reduction in cell apoptosis. The aforementioned drug combination was found to increase the level of ROS, which in turn mediates the increased level of apoptosis in cervical cancer cells.

However, further research and clinical trials are needed to fully assess the safety and efficacy of using LBH589 and AM1241 in the treatment of cervical cancer. Additionally, it's important to consider individual patient factors and potential side effects before implementing a new treatment strategy.

In conclusion, our study proved that LBH589 combined with AM1241 induced the apoptosis of cervical cancer cells by activating the ER stress pathway, DDR pathway and autophagy pathway. The administration of LBH589 combined with AM1241 promoted the activation of autophagy induced by LBH589, and increased ROS expression, which further promoted the apoptosis oxidative damage of cervical cancer cells. Our research provides a glimmer of possibilities for the use of LBH589 combined with AM1241 in the clinical treatment of cervical cancer.

### Supplementary Information

**Additional file 1:** **Figure S1.** Synergistic effect chart of AM1241 and LBH589 combined treatment on SiHa cervical cancer cells. Synergistic effect charts of SiHa cervical cancer cells were calculated using (A) ZIP, (B) HSA, (C) Bliss, and (D) Loewe reference models. These data were obtained using the SynergyFinder software. The synergistic effect is manifested as a ZIP synergy score of more than 1, and Loewe, Bliss, and HSA synergistic scores of more than 0 (*n*=5).

## Data Availability

The data used to support the findings of this study are available from the corresponding author upon request.

## References

[1] Arbyn M, Weiderpass E, Bruni L, de Sanjose S, Saraiya M, Ferlay J (2020). Estimates of incidence and mortality of cervical cancer in 2018: a worldwide analysis. Lancet Glob Health.

[2] Fang J, Zhang H, Jin S (2014). Epigenetics and cervical cancer: from pathogenesis to therapy. Tumour Biol.

[3] Duenas-Gonzalez A, Lizano M, Candelaria M, Cetina L, Arce C, Cervera E (2005). Epigenetics of cervical cancer. An overview and therapeutic perspectives. Mol Cancer.

[4] de Freitas NL, Deberaldini MG, Gomes D, Pavan AR, Sousa A, dos Santos JL (2020). Histone deacetylase inhibitors as therapeutic interventions on cervical cancer induced by human papillomavirus. Front Cell Dev Biol.

[5] Song X, Wang J, Zheng T, Song R, Liang Y, Bhatta N (2013). LBH589 Inhibits proliferation and metastasis of hepatocellular carcinoma via inhibition of gankyrin/STAT3/Akt pathway. Mol Cancer.

[6] Garrett LA, Growdon WB, Rueda BR, Foster R (2016). Influence of a novel histone deacetylase inhibitor panobinostat (LBH589) on the growth of ovarian cancer. J Ovarian Res.

[7] Qin G, Li Y, Xu X, Wang X, Zhang K, Tang Y (2019). Panobinostat (LBH589) inhibits Wnt/beta-catenin signaling pathway via upregulating APCL expression in breast cancer. Cell Signal.

[8] Liu B, Zhai J, Wang W, Liu T, Liu C, Zhu X (2022). Identification of tumor microenvironment and DNA methylation-related prognostic signature for predicting clinical outcomes and therapeutic responses in cervical cancer. Front Mol Biosci.

[9] Wasim L, Chopra M (2018). Synergistic anticancer effect of panobinostat and topoisomerase inhibitors through ROS generation and intrinsic apoptotic pathway induction in cervical cancer cells. Cell Oncol (Dordr).

[10] Yan L, Li J, Zhao T, Wang H, Lai G (2015). Over-expression of cannabinoid receptor 2 induces the apoptosis of cervical carcinoma Caski cells. Xi Bao Yu Fen Zi Mian Yi Xue Za Zhi.

[11] Mahmoud HM, Osman M, Elshabrawy O, Abdallah HMI, Khairallah A (2019). AM-1241 CB2 receptor agonist attenuates inflammation, apoptosis and stimulate progenitor cells in bile duct ligated rats. Open Access Maced J Med Sci.

[12] Ianevski A, Giri AK, Aittokallio T (2020). SynergyFinder 2.0: visual analytics of multi-drug combination synergies. Nucleic Acids Res.

[13] Russo M, Russo GL (2018). Autophagy inducers in cancer. Biochem Pharmacol.

[14] Liu S, Wang H, Mu J, Wang H, Peng Y, Li Q (2020). MiRNA-211 triggers an autophagy-dependent apoptosis in cervical cancer cells: regulation of Bcl-2. Naunyn Schmiedebergs Arch Pharmacol.

[15] Guo X, Xiao H, Guo S, Li J, Wang Y, Chen J (2019). Long noncoding RNA HOTAIR knockdown inhibits autophagy and epithelial-mesenchymal transition through the Wnt signaling pathway in radioresistant human cervical cancer HeLa cells. J Cell Physiol.

[16] Zhou Q, Dong J, Luo R, Zhou X, Wang J, Chen F (2019). MicroRNA-20a regulates cell proliferation, apoptosis and autophagy by targeting thrombospondin 2 in cervical cancer. Eur J Pharmacol.

[17] Di Fazio P, Waldegger P, Jabari S, Lingelbach S, Montalbano R, Ocker M (2016). Autophagy-related cell death by pan-histone deacetylase inhibition in liver cancer. Oncotarget.

[18] Mao Y, Huang Y, Zhang Y, Wang C, Wu H, Tian X (2019). Cannabinoid receptor 2selective agonist JWH015 attenuates bone cancer pain through the amelioration of impaired autophagy flux induced by inflammatory mediators in the spinal cord. Mol Med Rep.

[19] Liu W, Chen C, Gu X, Zhang L, Mao X, Chen Z (2021). AM1241 alleviates myocardial ischemia-reperfusion injury in rats by enhancing Pink1/Parkin-mediated autophagy. Life Sci.

[20] Oakes SA, Papa FR (2015). The role of endoplasmic reticulum stress in human pathology. Annu Rev Pathol.

[21] Zhang Y, Bai C, Lu D, Wu X, Gao L, Zhang W (2016). Endoplasmic reticulum stress and autophagy participate in apoptosis induced by bortezomib in cervical cancer cells. Biotechnol Lett.

[22] Hu L, Wang H, Huang L, Zhao Y, Wang J (2016). Crosstalk between autophagy and intracellular radiation response (Review). Int J Oncol.

[23] Hanahan D, Weinberg RA (2011). Hallmarks of cancer: the next generation. Cell.

[24] Spriggs CC, Blanco LZ, Maniar KP, Laimins LA (2019). Expression of HPV-induced DNA Damage Repair Factors Correlates With CIN Progression. Int J Gynecol Pathol.

[25] Li L, Tan J, Miao Y, Lei P, Zhang Q (2015). ROS and Autophagy: Interactions and Molecular Regulatory Mechanisms. Cell Mol Neurobiol.

