# Supplementary figures and images for: Panobinostat (LBH589) combined with AM1241 induces cervical cancer cell apoptosis through autophagy pathway
Source: BMC Pharmacol Toxicol. 2023 Sep 22;24:45. doi: 10.1186/s40360-023-00686-7 (PMC10517494; doi:10.1186/s40360-023-00686-7)

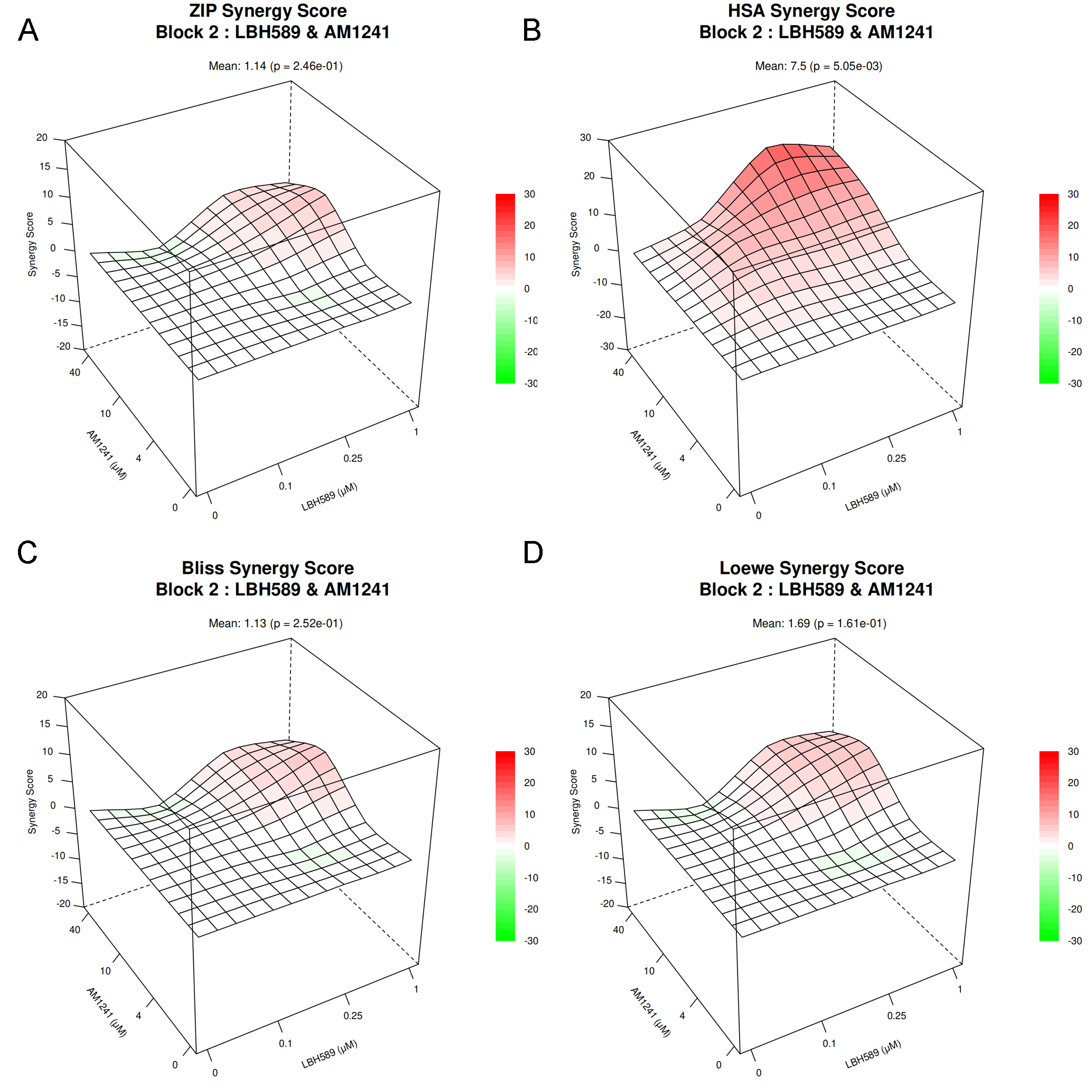

Supplement: Supplementary file 1 — Additional file 1: Figure S1. Synergistic effect chart of AM1241 and LBH589 combined treatment on SiHa cervical cancer cells. Synergistic effect charts of SiHa cervical cancer cells were calculated using (A) ZIP, (B) HSA, (C) Bliss, and (D) Loewe reference models. These data were obtained using the SynergyFinder software. The synergistic effect is manifested as a ZIP synergy score of more than 1, and Loewe, Bliss, and HSA synergistic scores of more than 0 (n=5). [file 40360_2023_686_MOESM1_ESM.tif]
